# Applications of the Amniotic Membrane in Vitreoretinal Surgery

**DOI:** 10.3390/jcm9082675

**Published:** 2020-08-18

**Authors:** Tomaso Caporossi, Ruggero Tartaro, Daniela Bacherini, Bianca Pacini, Lorenzo De Angelis, Lorenzo Governatori, Laura Di Leo, Leandro Oliverio, Stanislao Rizzo

**Affiliations:** 1 Department of NEUROFARBA, Ophthalmology, University of Florence, Careggi, 50134 Florence, Italy; ruggero.tartaro@gmail.com (R.T.); daniela.bacherini@unifi.it (D.B.); pacinibianca@gmail.com (B.P.); lor.deangelis89@gmail.com (L.D.A.); lorenzo.gov@gmail.com (L.G.); laura.dileo82@gmail.com (L.D.L.); leandro.oliverio92@gmail.com (L.O.); 2 Department of Ophthalmology, Catholic University of Sacred Hearth-Foundation “Policlinico Universitario A. Gemelli”-IRCCS, 00168 Rome, Italy; stanislao.rizzo@gmail.com

**Keywords:** vitreoretinal surgery, amniotic membrane, vitrectomy, macular hole, retinal detachment, age related macular degeneration, optic pit maculopathy

## Abstract

Recently, the use of the human amniotic membrane (hAM) has been extended to treat retinal disorders, such as macular holes that failed to close and retinal tears. The hAM has demonstrated the induction of a recovery process of the external retinal layers involving the external limiting membrane (ELM) and the ellipsoid zone (EZ). After that, the application of the hAM for retinal pathologies was extended to large macular tears, high myopic retinal detachment associated with MH, paravascular tears, serous macular detachment associated with optic pit, complicated retinal detachment and advanced age-related macular degeneration (AMD). The hAM has shown a potential in repairing retinal tissue through a regeneration process. This review aims to highlight the use of the hAM in various vitreo-retinal surgical fields, and to confront it with other cutting-edge surgical techniques used to treat challenging vitreo-retinal pathologies.

## 1. Introduction

The human amniotic membrane (hAM), also known as amnion, envelopes the human fetus. It is a semitransparent sheet and its thickness is about 0.02–0.05 mm. It is characterized by a epithelium, a basement membrane, a compact layer, a fibroblast layer and a spongy layer [1,2,3,4]. The amniotic membrane has been used in several fields of the eye surgery, such as the covering of corneal ulcers and the reconstruction of the conjunctiva. The amniotic membrane showed excellent anti-angiogenic and anti-microbial properties and poor immunogenicity [5,6,7,8,9,10,11,12]. The hAM can secrete a variety of cytokines including: transforming growth factor (TGFα, TGFβ-1, β-2, and β-3), basic fibroblast growth factor, epithelial growth factor, the hepatocyte growth factor and its receptor and the keratinocyte growth factor and its receptor [13,14,15,16,17,18].

In addition, the amniotic membrane can reconstruct surgically sectioned nerves in the animal model [19,20,21].

The hAM epithelium produces brain natriuretic peptides and hormones that release corticotropin, a cell proliferation promoter [22,23]. The hAM wraps, furthermore, if surgically implanted are a reservoir of neurotrophic factors, such as the nerve growth factor (NGF), brain-derived neurotrophic factor (BDNF), neurotrophin 3 (NT-3), glial cell-derived neurotrophic factor (GDNF) and the ciliary neurotrophic factor (CNTF) [24,25,26].

Some authors have also grown human retinal pigmented epithelium (RPE) cells, in vitro, on a layer of hAM. Here, the cells have proliferated effectively forming an organized mono layer [27]. Furthermore, Ohno-Matsui et al. [28] have shown that RPE cells grown on a hAM sheet do not only organize in a single layer, but secrete numerous growth factors. In addition, a hAM sheet was implanted into the sub-retinal space in pigs with a iatrogenic damage to the retinal pigmented epithelium. The hAM, after the implant, promoted a reduction of the iatrogenic damage and was covered by a mono-layer of epithelial cells [29].

The application of the hAM in vivo for vitreo-retinal pathologies is more recent, and began with macular holes that failed to close and retinal tears [30]. The hAM has demonstrated the induction of a recovery process involving the external retinal layers such as the external limiting membrane (ELM) and the ellipsoid zone (EZ).

The hAM application for retinal pathologies was extended to treat large macular tears [31], high myopic retinal detachment associated with MH [32], paravascular tears [33], serous macular detachment associated with optic pits [34], complicated retinal detachment [30] and advanced age related macular degeneration (AMD) [35].

This chapter resumes the application of the hAM in vitreoretinal pathologies.

## 2. Materials and Methods

This chapter highlights the use of the hAM in various vitreo-retinal surgical fields, and confronts it with other cutting-edge surgical techniques used to treat challenging vitreo-retinal pathologies.

### Amniotic Membrane to Treat Macular Holes, Surgical Technique

We used a cryopreserved hAM delivered from the eye bank in Lucca, Italy or the eye bank in Mestre, Italy.

When we started performing the hAM transplant, the technique involved the intraocular trimming of the hAM plug to adjust its final dimensions. Then we found more accurate and less traumatic the use of a disposable cutaneous punch (Disposable Biopsy Punch, Kai Medical, Solingen, Germany) to regulate the size of the hAM patch. The structural pre-operative optical coherence tomography (OCT) was used to determine the MH diameter and therefore the hAM patch dimension.

We chose a 1 mm, 1.5 mm and 2 mm for macular holes, 3 and 5 mm for exudative and atrophic age-related macular degeneration and 1.5 mm optic pit cases. The plug should have the right diameter because an hAM disc that is larger than the basal diameter of the macular hole may corrugate and halt the anatomical and functional recovery. On the other side, a too short diameter of the AM plug will not induce the MH resolution.

The macular hole should be prepared to contain the hAM plug. This is performed using an illuminated PIK (Alcon PIK Endoilluminator, Alcon Fort Worth, TX, US), or a silicone tip backflush, to gradually detach the MH edges from the RPE. However, particular care must be taken not to damage the RPE.

At the beginning of our experience, we introduced the hAM plug through a 23-gauge trocar, inside the vitreous chamber, using 27-gauge forceps. Plugs of more than 3 mm disc diameter needed a 20-gauge sclerotomy for their intraocular insertion. Using this technique, we observed a hAM wrinkling in some cases, therefore we introduced the DSAEK-inspired insertion technique [36]. The hAM plug was contained in a plastic support and maintained at the entrance of the nasal trocar. Vitreoretinal forceps are passed from the temporal trocar to the nasal trocar and the hAM disc was dragged inside the vitreous chamber. This technique avoids the hAM excessive manipulation and therefore deterioration, which may result in a lower adhesion of the retina to the RPE.

The chorion layer of the hAM, once inside the vitreous chamber, should be recognized because it needs to face the RPE in order to obtain a firm adhesion. It is useful to know that the chorion tends to remain adherent to the vitreo-retinal forceps. Hence by re-grabbing the plug we can have the chorion layer in contact with the forceps. The hAM is inserted inside the MH using a PIK or a flexible laser tip and if the chorion layer will face the RPE the plug will remain adherent during the fluid-air exchange.

**Figure 1:**
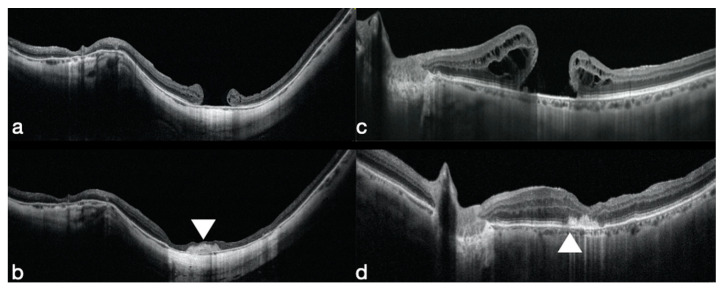
(**a–c**) preoperative SD-OCT (spectral domain optical coherence tomography) showing a failed high myopic macular hole and a failed non-high myopic macular hole. (**b–d**) 6-months OCT (optical coherence tomography) shows in both cases the human amniotic membrane (hAM) patch well positioned under the retina (white arrows) and the macular hole closed. A partial differentiation of the external retinal layers such as the external limiting membrane and the ellipsoid zone is evident (**d**).

Rizzo et al. treated the first eight cases, affected by a MH that failed to close, using a hAM plug, and obtained a 100% closure rate and an average BCVA improvement from 20/800 to 20/50 [30] (Figure 1c,d).

## 3. Outcomes of the Human Amniotic Membrane Retinal Transplant and Comparison with Other Cutting Edge Vitreoretinal Surgical Techniques

### 3.1. Macular Holes that Failed to Close

Macular holes that failed to close are still a challenge for vitreoretinal surgeons. The incidence varies from 0 to 39% [37,38], without internal limiting membrane (ILM) peeling and from 0% to 8.6% in eyes in which the ILM has been peeled off [39]. Many modern surgical options have been proposed to treat this condition such as the introduction of autologous fragments from the anterior lens capsule, autologous neuroretina or autologous ILM plugs into the macular hole [40,41,42,43,44]. These techniques aim to produce a sealing of the hole with variable visual acuity (VA) recovery.

The application of the human amniotic membrane (hAM) plug transplanted into the sub-retinal space to treat macular holes that failed to close, has shown an improvement of the anatomical and functional results.

Rizzo et al. treated the first eight cases, affected by a MH that failed to close, using a hAM plug, and obtained a 100% closure rate and a average BCVA improvement from 20/800 to 20/50 [30] (Figure 1c,d).

Morizane et al. treated 10 recurrent MHs using an autologous ILM transplant and obtained a 90% closure rate [40]. Grewal and Mahmoud in 2015 described the autologous neurosensory retinal transplantations for recurrent HMMH [42]. The same authors, in a multicenter study, treated 41 eyes of 41 patients affected by a recurrent MH, using autologous retinal transplant, and they reported a MH closure in 36 eyes out of 41 (87.8%) [45].

In high myopic eyes affected by an unresolved macular hole, which already underwent a PPV with ILM peeling, several techniques have been described, such as the use of anterior and posterior lens capsular flap transplantation [43], an autologous ILM transplantation [40], autologous neurosensory retinal free flap transplantation (ANRFF) with gas or silicone oil tamponade [42] and ILM or ANRFF transplantation with the addition of autologous blood [46,47,48].

Caporossi et al. treated 16 eyes of 16 patients affected by high myopic MH without retinal detachment, using an hAM plug. Fifteen out of 16 eyes closed with one surgery (93.75%) and 100% with two surgeries. The best corrected visual acuity improved from 20/200 to 20/100. Eleven patients (68.75%) had a BCVA improvement; four patients (25%) had no BCVA improvement and one patient had a VA worsening. Ten patients had 20% SF_6_, as post-operative tamponade, and six had air, no differences in the final BCVA were observed. (Figure 1a,b)

Morizane et al. [40] proposed to harvest extra-foveal ILM remnants and transplant them into the macular hole area. They reported, in a case series of 10 patients, two cases of recurrent HMMH treated with autologous ILM transplantation. They showed a 100% MH closure rate [40].

Chen published six recurrent HMMH of 20 patients treated with capsular lens fragments transplantation with a 100% closure rate and a final mean BCVA of 0.8 logMAR (20/125) [43]. Peng et al. treated 10 cases of HMMH with lens capsular flap transplantation (LCFT) and autologous whole blood application with only one unsuccessful case (10%), and a final BCVA of 1.34 logMAR (20/450) [44].

We may argue that the hAM plug transplant is an easier technique than the retina or the ILM free flap and Rizzo, Caporossi and colleagues did not find any sign of rejection. They reported an extremely high closure rate, and also reported interesting findings in terms of retinal regeneration.

### 3.2. High Myopic Macular Hole Associated with Retinal Detachment

High myopic macular hole-induced retinal detachment (HMMH RD) often causes severe visual impairment; it occurs mainly in people with highly myopic eyes who have a posterior staphyloma [49,50].

In 2019 Caporossi et al. treated 10 patients affected by HMMH RD, using the hAM, with a 100% macular hole closure and retina reattachment rate. The BCVA improved from 20/1000 to 20/160. Five patients were tamponed with standard silicone oil and five with 16% C_3_F_8_. No statistically significant difference was reported between the two groups.

Lai et al. in their case series with autologous blood for HMMH RD enrolled 27 patients and obtained a final closure rate of 96% after one surgery [46].

In 2018 Chen et al. proposed the free ILM flap, and achieved a 100% closure rate and a retinal re-attachment in 13 patients [51].

The hAM transplant is easier than the ILM free flap technique, because there is no need to harvest ILM flaps in a myopic eye. This is relatively challenging, and there is also a risk of iatrogenic retinal tears. Conversely, the hAM, after the intraocular insertion, can be directly positioned into the macular hole. The results, presented by Prof Rizzo and Caporossi, are very encouraging and showed a 100% reattachment rate with also a promising integration of the hAM, that generated an external retinal layer reconstruction.

### 3.3. Complicated Retinal Detachment

In 2019, Rizzo et al. [30] treated six cases of recurrent retinal detachment complicated with PVR, using a hAM implant inside the retinal breaks. All the cases were tamponed using silicone oil, that was removed after 3 months. All the cases achieved a retinal reattachment with a BCVA improvement from 20/2000 to 20/125. In all the cases, the post-operative OCT showed the integration of the hAM patch inside the retinal breaks, and a thin layer of tissue growing over the patch. In all the cases no laser retinopexy was applied.

More recently Caporossi et al. [31] treated two cases of retinal detachment with a large macular tear using a hAM patch positioned under the retina, no laser and silicone oil as endotamponade were applied. In both cases the BCVA improved from light perception to 20/400 and a 3-month follow-up OCT showed a partial regrowth of the retinal layers in the macular area that covered the site of the previous retinal tear.

Retinal detachment after PPV for myopic foveoschisis is a rare complication, and is often associated with macular holes or posterior pole breaks. These breaks are often localized close to vessels, over areas of patchy chorioretinal atrophy. The endophotocoagulation is not possible because these are atrophic areas, and the trans-scleral cryotherapy is not indicated because these lesions are at the posterior pole [52,53,54]. Differently, an ILM plug transplantation can stimulate the posterior-pole retinal breaks closure even without a laser retinopexy [55]. In the case of extended ILM peeling during the first surgery, the ILM remnants are difficult to harvest, Caporossi et al. proposed a hAM patch implant inside the retinal breaks. They reported two cases out of two of retinal reattachment. The BCVA improved from 20/2000 to 20/250 [33].

### 3.4. Optic Disk Pit Associated Macular Detachment

Serous retinal detachment (ODP-M) is a frequent complication of the optic pit (25–75%) and it is associated with a bad visual acuity prognosis [56,57,58].

**Figure 2:**
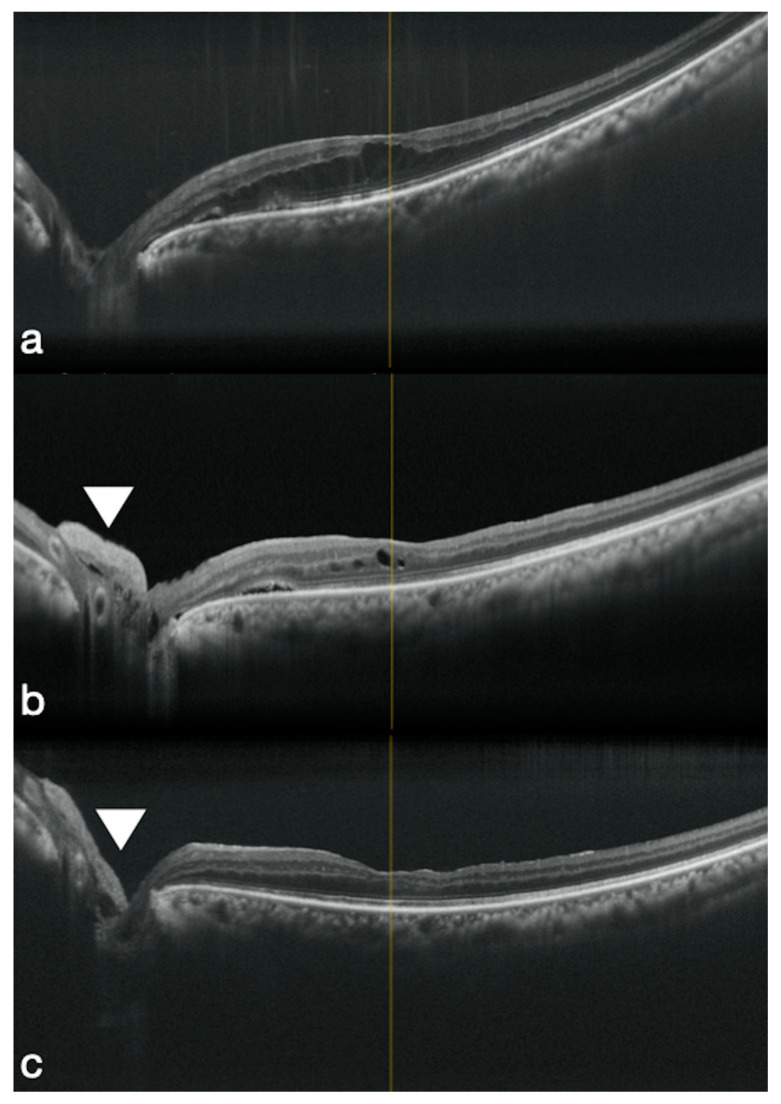
Preoperative OCT shows an optic disc pit maculopathy (**a**). 1 and 3 months postoperative OCT shows the hAM patch positioned inside the optic disc area (white arrows) and the macular schisis completely solved (**b**,**c**).

Rizzo et al. proposed three cases of ODP-M treated using an hAM plug positioned inside the optic disc pit. The sub-retinal fluid reduced during the first 6 months after surgery and the average visual acuity improved from 20/40 to 25/20 at the 6th month after surgery [34] (Figure 2).

García-Arumí et al. [59] treated 11 patients with PPV, laser and gas as endotamponade. They achieved the anatomical resolution and a significant visual improvement in all but 2 cases. Hirakata et al. [60] achieved the complete fluid resolution in 11 ODP-M patients treated with PPV, PVD induction and gas tamponade without laser. In 2012, Hirakata reported a series of seven patients treated with PPV and PVD induction without laser nor gas tamponade, the BCVA improved but in these cases the sub-retinal fluid did not resolve completely.

Recently, Mohammad et al. [61] proposed the use of the ILM autologous transplant into the optic pit and showed a faster sub-retinal fluid reabsorption than the standard technique.

The autologous scleral transplantation into the optic pit is an alternative technique, although the complete subretinal and intraretinal fluid reabsorption were achieved in 12 months after surgery in all the three cases treated using this technique [62].

Parag et al. [63] reported two cases operated on using a scleral autograft; they reported a complete sub-retinal fluid reabsorption after 1 year.

Rizzo and Caporossi et al. showed the usefulness and safeness of the hAM to treat ODP-M. The hAM, after the intraocular insertion, can be directly positioned into the optic disc pit. The results, presented by Prof Rizzo and Caporossi, are very encouraging, and showed a 100% resolution of the sub-retinal fluid.

### 3.5. Age-Related Macular Degeneration

End-stage age-related macular degeneration is the leading cause of blindness in the industrialized world and Prof Rizzo’s group for the first time introduced the hAM in the treatment of this degenerative chronic disease [35].

A 41-gauge needle was mounted on a 10-cc syringe containing balancing saline solution (BSS) which was injected in the sub-retinal space and three to four air-fluid exchanges were carried out to provoke a retinal detachment.

In the case of choroidal neovascular lesions (CNV), a 180° retinectomy was created in the temporal retinal periphery, in order to remove the neovascular tissue, which was aspirated using the vitrectomy probe. In the cases of geographic atrophy, the detachment was created at the posterior pole and the retina was delicately divided from the atrophic scar using a cannula or an endoilluminated PIC, introduced through a retinectomy, created near the vascular arcades.

A hAM sheet was positioned in the macular area with the chorion facing the RPE. The retina was re-attached over the hAM patch with the aid of perfluorocarbon, and an endolaser retinopexy was carried out at the edge of the retinectomy.

A perfluorocarbon-silicon oil direct exchange was performed at the end of the surgery. In all the cases a standard silicon oil 1000 cSt (Oxane 1300 Bausch + Lomb Incorporated, Rochester, NY, USA) was used as endotamponade. The patients maintained a face-down position for 7 days. The silicone oil was removed 4 months after the operation in all the cases without any complication.

In the hemorrhagic group (six patients), the initial mean BCVA was 2 logMAR (20/2000 Snellen), and the final mean BCVA was 1.1 logMAR (20/250 Snellen) *p* = 0.00042.

In the geographic atrophy group (five patients), mean BCVA improved from 1.84 logMAR (20/2000 Snellen), range 20/2000–20/800 (2–1.6 logMAR), to 1.26 logMAR (20/320 Snellen), range 20/2000–20/100 (2–0.7 logMAR) *p* = 0.0084.

Adaptive optics (AO) analyses were carried out over the retinal areas where the highest functionality was identified, using the microperimetry analysis. The images showed a photoreceptors presence at the hAM edges, which was confirmed by the built-in software.

**Figure 3:**
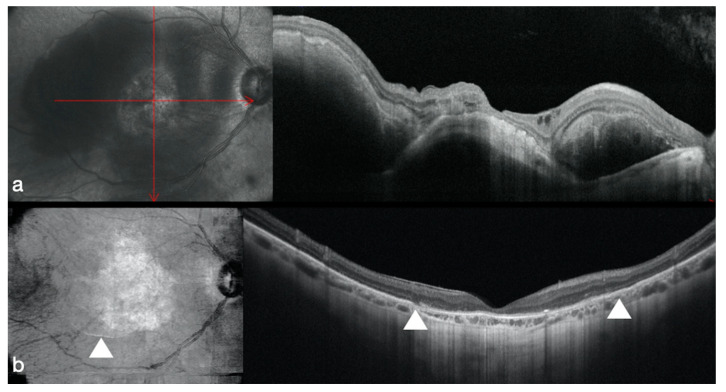
Preoperative OCT showing a sub macular hemorrhage involving the posterior pole (**a**) 6 months postoperative OCT shows a sub-retinal hAM patch positioned at the posterior pole (white arrows). We can recognize the outer retinal layers such as the external limiting membrane and ellipsoid zone (**b**).

The better post-operative sensibility responses were registered, using microperimetry, at the edge of the amniotic membrane patch in 10 out of 11 patients, the ones with the better visual recovery (>1.3 logMAR) (Figure 3).

In recent times, interesting new surgical treatments have been suggested for end-stage AMD. Autologous retinal and chorioretinal transplants have been proposed by Dr Parolini [64]. However, the autologous choroidal and retinal transplants are technically challenging procedures with a high risk of intra-operative and post-operative complications, such as retinal detachment with proliferative vitreoretinopathy. The hAM, on the other hand, is easier to insert into the subretinal space than a fragile autologous retinal or chorioretinal transplant. The excessive manipulation of retinal and chorioretinal flaps can lead to tissue disorganization with a tangible impact on the visual funtion. Prof. Da Cruz [65] created an embryonic stem cell-derived retinal pigment epithelium patch. The main problem is that the embryonic stem cell-derived retinal pigment epithelium patch was associated with systemic corticosteroid therapy because of the risk of immunogenic graft rejection. Conversely, the hAM sheet, implanted in the sub retinal space, did not allow any immunologic reaction. Prof Rizzo and Dr Caporossi think that the hAM may be a step forward in the management of the AMD.

## 4. Conclusions

In the recent years, the application of the hAM for retinal pathologies has been widely investigated.

The ability to be integrated in the retinal tissue without immunologic reactions or major postoperative complications, make it a useful basal membrane, and represent an interesting approach to different retinal pathologies.

Further prospective, larger, randomized controlled studies are needed to confirm our results.
